# Telemedicine platform for health assessment remotely by an integrated nanoarchitectonics FePS_3_/rGO and Ti_3_C_2_-based wearable device

**DOI:** 10.1038/s41528-022-00208-1

**Published:** 2022-08-15

**Authors:** Jayraj V. Vaghasiya, Carmen C. Mayorga-Martinez, Martin Pumera

**Affiliations:** 1 Center for Advanced Functional Nanorobots, Department of Inorganic Chemistry, Faculty of Chemical Technology, University of Chemistry and Technology Prague, Technická 5, 166 28 Prague, Czech Republic; 2 Energy Research Institute@NTU (ERI@N), Research Techno Plaza, X-Frontier Block, Level 5, 50 Nanyang Drive, 637553 Singapore, Singapore; 3 Faculty of Electrical Engineering and Computer Science, VSB - Technical University of Ostrava, 17. listopadu 2172/15, 70800 Ostrava, Czech Republic; 4 Department of Medical Research, China Medical, University Hospital, China Medical University, No. 91 Hsueh-Shih Road, Taichung, 40402 Taiwan

**Keywords:** Electronic properties and materials, Electronic properties and devices

## Abstract

Due to the emergence of various new infectious (viral/bacteria) diseases, the remote surveillance of infected persons has become most important, especially if hospitals need to isolate infected patients to prevent the spreading of pathogens to health care personnel. Therefore, we develop a remote health monitoring system by integrating a stretchable asymmetric supercapacitor (SASC) as a portable power source with sensors that can monitor the human physical health condition in real-time and remotely. An abnormal body temperature and breathing rate could indicate a person’s sickness/infection status. Here we integrated FePS_3_@graphene-based strain sensor and SASC into an all-in-one textile system and wrapped it around the abdomen to continuously monitor the breathing cycle of the person. The real body temperature was recorded by integrating the temperature sensor with the SASC. The proposed system recorded physiological parameters in real-time and when monitored remotely could be employed as a screening tool for monitoring pathogen infection status.

## Introduction

Many infectious diseases are connected with several physiological changes that can be monitored using wearable health sensors^1,2^. An abnormal body temperature, breathing rate, and diastolic blood pressure could indicate a patient’s infection status. Recently the world faced a pandemic by SARS-CoV-2 virus and many health care personnel were infected by the direct contact with infected patients during their physiological parameters assessment. Demonstrating that it is necessary to maintain social distance and quarantine to decrease the risk of spreading the infection to others. In this situation, a remote health monitoring system (RHMS) has proven to be the safest method for health monitoring of infected patients^3,4^. RHMS can be defined as the delivery of healthcare services over a distance. RHMS typically comprises three main components: a sensing device, data transmission device, and power device^5^. The sensing device detects various physiological parameters like body temperature, heart rate, and blood pressure, and converts them into electrical signals that are transmitted *via* a wireless connection to a smartphone or the cloud for further analysis. Both the sensing and data transfer processes necessitate the use of an energy storage source.

In comparison to other typical electronic gadgets, a wearable health monitoring system has unique power source requirements^6^. First, the energy storage device as well as the sensing device must be stretchable, mechanically and electrically stable to enable a conformable shape with the body for constant detection performance. Second, the device often comes into contact with human body parts, thus, the energy storage and sensing devices must be biocompatible to minimize undesirable immune responses and other negative effects. With regard to the above points, we fabricated a proof-of-concept wearable integrated healthcare monitoring system that employs a stretchable asymmetric supercapacitor (SASC) and strain sensor. Where SASC was fabricated using two different types of 2D materials such as transition metal carbides (e.g., Ti_3_C_2_) and metal phosphorus chalcogen@ reduced graphene oxide composite (e.g., FePS_3_@rGO). While strain sensor was assembled using the same FePS_3_@rGO composite.

Emerging family of layered materials, metal phosphorous chalcogenides with a common formula of MPX_y_ where M represents a transition metals, X presents a chalcogen (S or Se) and can be 3 or 4 possess superior electronic, magnetic and anisotropy properties. They have been applied in a wide variety of applications such as optoelectronics, spintronic, sensing, hydrogen adsorption, catalysis as well as batteries electrochemical properties^7–11^. Recently, considerable attention has been focused on utilizing MPS_3_ (M = Iron (Fe), Nickel (Ni), tin (Sn), cobalt (Co), etc.) materials for lithium-ion batteries^12–14^. Specifically, FePS_3_, NiPS_3_, and MnPS_3_ have been identified as promising cathode materials in batteries, with greater capacity and longer operational periods than the carbon and metal oxide electrodes investigated previously^13–15^. Moreover, in our earlier study, we stated that some of the MPS_3_ materials (e.g., FePS_3_, NiPS_3_, and CoPS_3_) showed remarkable performance as electrocatalysts for hydrogen and oxygen production^16^. However, MPX_y_ has not yet received much interest as a flexible and portable energy source for wearable electronic applications. On the other hand, the Ti_3_C_2_T_x_ (MXenes) are one of the most frequently researched owing to their inexpensive cost and range of benefits^17–20^. Therefore, Ti_3_C_2_T_x_ can be easily incorporated into fabrics, showing promise in health sensors and energy storage applications^21–23^.

**Fig. 1:**
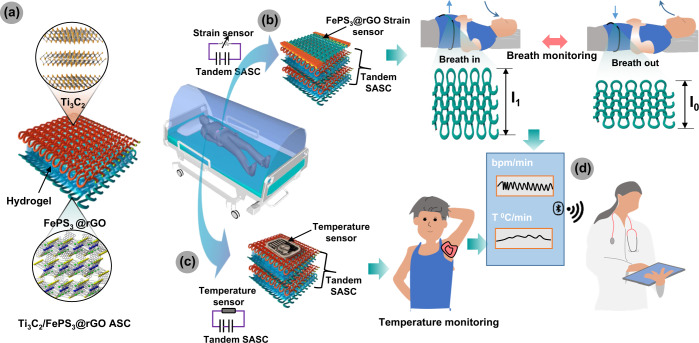
Telemedicine platform for remotely health monitoring by integrated 2D FePS_3_@rGO and Ti_3_C_2_ based wearable devices. **a** Schematic illustration of the Ti_3_C_2_/FePS_3_@rGO SASC. **b** Integrated FePS_3_@rGO-based strain sensor with series-connected two SASC for real-time breath monitoring. **c** Integrated temperature sensor with series-connected two SASC for real-time body temperature monitoring. **d** Bluetooth or Wi-Fi signal sources used to transfer breathing and temperature data to a healthcare provider.

In this study, we report on the fabrication of a textile-based remote health monitoring system comprising a high-performance stretchable asymmetric supercapacitor (SASC) and strain sensor (Fig. 1). A composite based on FePS_3_ and rGO was used as an electrode of SASC and strain sensor coated on stretchable fabric. The second electrode of SASC is based on Ti_3_C_2_T_x._ This unique integrated system can be attached directly to the human body (abdomen) to monitor accurately a patient’s breathing rate. In addition, SASC was used to power temperature sensor attached to the armpit and monitor the body temperature as well. This system enables patients to keep track of these health indicators without having direct contact with health care personnel. Most importantly, the data from real-time monitoring of breathing and body temperature can be sent *via* wireless communication to the hospital cloud system for clinical assessment.

## Result and discussion

Remotely healthcare system may become a basic necessity for monitoring patients with infections illnesses (e.g., SARS-CoV-2), especially when they are under quarantine. Thus, the purpose of this work was to develop a lightweight and wearable health monitoring system that could monitor critical physiological parameters during all stages of infection diseases. Intending to design a remote health monitoring system, we integrated strain sensor and temperature sensor with series-connected two SASCs into all in one textile system. This all-in-one wearable health system represents a potential application for monitoring the physiological parameters of patients and wirelessly relaying data to the health care provider. The fabrication procedures for the SASC and strain sensor are schematically depicted in Supplementary Fig. 1 We assembled a fabric-based SASC by combining negative Ti_3_C_2_ and positive FePS_3_@rGO electrode in a polymeric hydrogel. The strain sensor is made of the same FePS_3_@rGO materials. Also, presented a scalable fast deposition process for spray-coating Ti_3_C_2_ and FePS_3_@rGO onto a wearable fabric substrate for SASC and strain sensor applications. To showcase a real-world application, we first must examine the morphology and electrochemical performance of the active 2D materials.

### Morphology characterization of Ti_3_C_2_/FePS_3_@rGO SASC

**Fig. 2:**
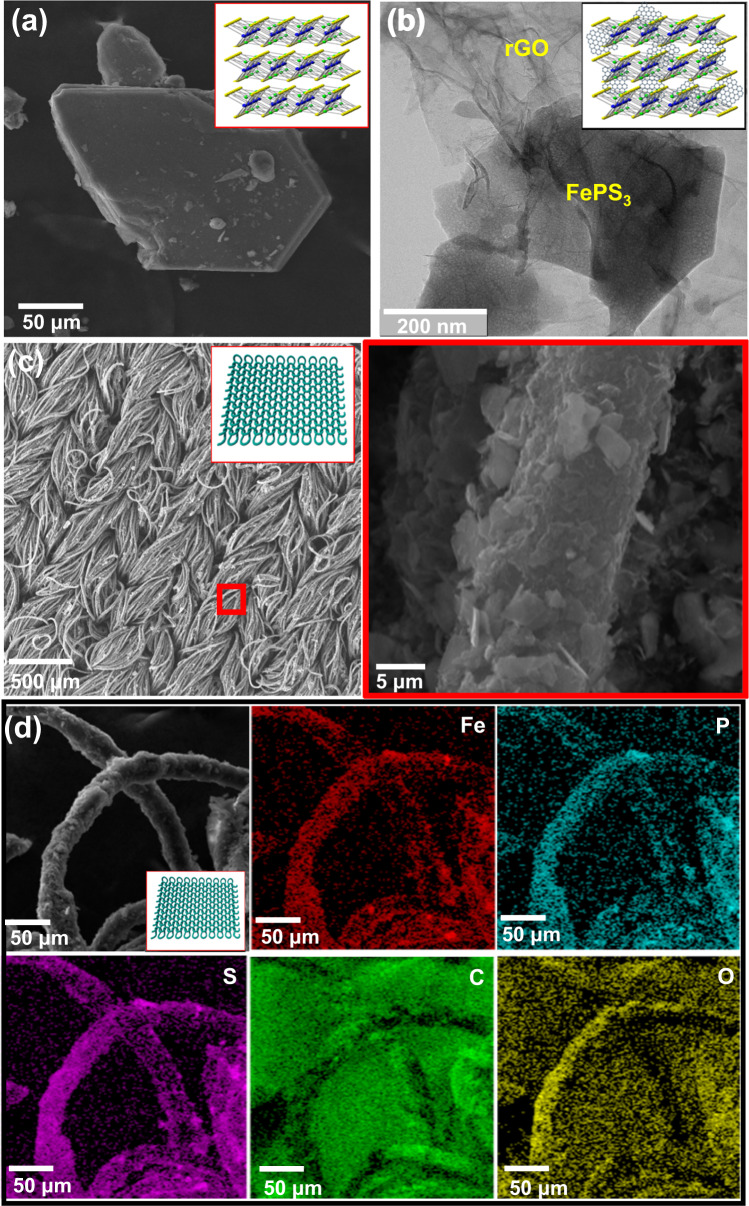
Morphology study of FePS_3_, FePS_3_@rGO and FePS_3_@rGO coated fabric. **a** SEM image of bulk FePS_3_. **b** TEM image of FePS_3_@rGO. **c** Low- and high-magnification SEM images of FePS_3_@rGO-coated fabric. **d** EDS mapping of FePS_3_@rGO-coated fabric.

A typical scanning electron microscopy (SEM) image of the bulk FePS_3_ exhibits a large polygonal stack (Fig. 2a), which is consistent with prior results^24^. To obtain FePS_3_ nanosheets, physical exfoliation was carried out using a strong sonication bath. After 2 h of sonication, a colloidal solution was obtained (Supplementary Fig. 2a). Figure 2b shows a transmission electron microscopy (TEM) image of FePS_3_@rGO colloidal solution (Supplementary Fig. 2b), where clear exfoliation is observed. Small FePS_3_ nanosheets are scattered on large rGO sheets, yielding a hybrid scaffold that can be seen by the contrast between two components. As revealed in SEM images of the FePS_3_@rGO-coated on cotton fabric (Fig. 2c), the nanosheets are uniformly and tightly wrapped around the entire fabric matrix. Figure 2c (right pannel), d shows a high-resolution SEM image of FePS_3_ coated fabric as well as the corresponding elemental mapping and energy-dispersive X-ray (EDS) spectrum (Supplementary Fig. 3a). The distribution of Fe, P, C, S, and O on the fabric surface is uniform, suggesting that the fabric is tightly wrapped by a layer of FePS_3_@rGO. The crystal structure of FePS_3_ is determined using X-ray diffraction (XRD). The XRD patterns of FePS_3_@rGO are shown in Supplementary Fig. 4a, with peak characteristics at 15.9°, 26.82°, 30.23°, 35.48°, 47.59°, and 55.49° on the 2θ scale corresponding to the (001), (002), (−201), (131), (202), and (−331) planes of FePS_3_ phase (1998-JCPDS 78–496)^25^. The sharpness of the different peaks indicates that FePS_3_ is highly crystalline^26^, meaning that FePS_3_ has no impurities. To investigate more about surface properties, nitrogen adsorption-desorption was measured for pristine FePS_3_ and FePS_3_@rGO composite (Supplementary Fig. 4b). The specific surface area (SSA) of pristine FePS_3_ is 12.6 m^2^ g^−1^ due to the serious restacking structure that obstructs accessibility of FePS_3_ to electrolyte ions. On the other hand, SSA of FePS_3_@rGO is obtained as 78 m^2^ g^−1^, which is significantly higher. This is due to the rGO having a large surface area on account of nanopores on the surface^27,28^. Furthermore, the introduction of rGO into the FePS_3_ can aid in the reduction of serious restacking and establish an inter-conductive path is suitable for accelerating transport and the diffusion of electrolyte ions in SC applications that require rapid charge-discharge cycles.

**Fig. 3:**
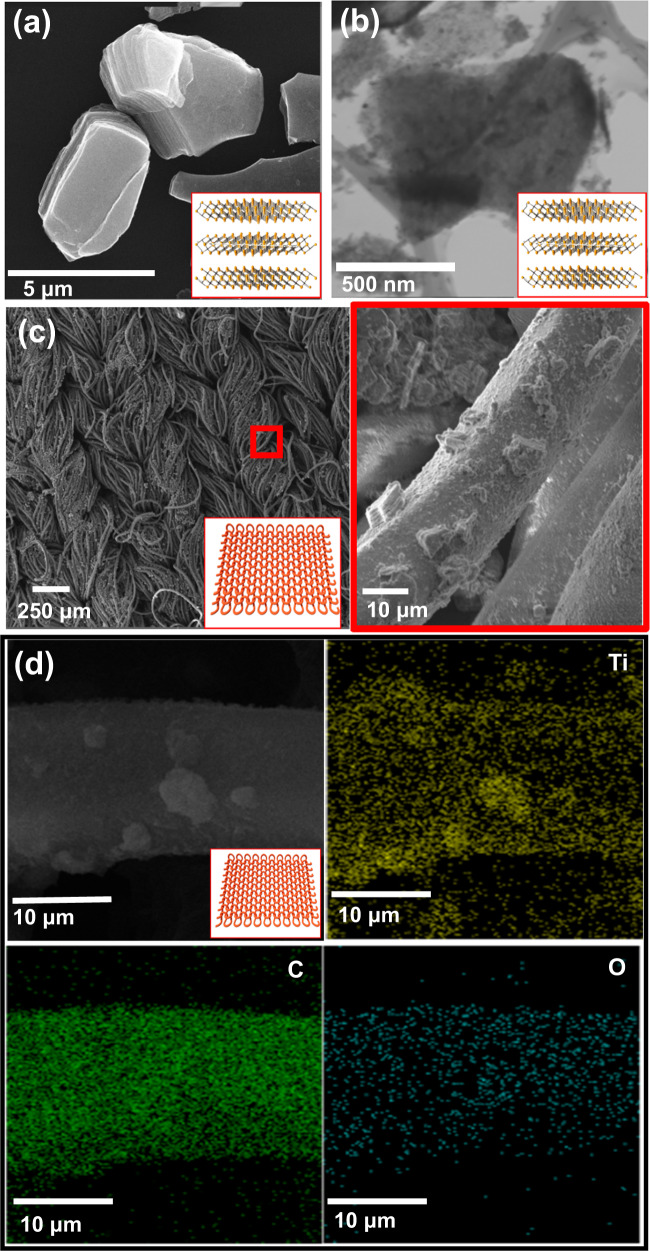
Morphology study of Ti_3_C_2_ and Ti_3_C_2_ coated fabric. **a**, **b** SEM and TEM images of Ti_3_C_2_. **c** low- and high-magnification SEM images of Ti_3_C_2_ coated fabric. **d** EDS mapping of Ti_3_C_2_ coated fabric.

Figure 3a displays the SEM image of pristine Ti_3_C_2_ with a multi-layered structure. These multi-layered structures intercalated with water molecules yielded a single layer Ti_3_C_2_ (Fig. 3b). As stated earlier, the exfoliated Ti_3_C_2_ can be easily dispersed in water due to a large number of oxygen-containing groups on the surface of Ti_3_C_2_ (Supplementary Fig. 2c)^29–31^. Thus, the homogeneous Ti_3_C_2_ dispersion shows excellent stability and typical colloidal nature, making it appropriate for a spray-coating technique (Fig. 3c). The majority of the Ti_3_C_2_ is deposited on the fabric surface without aggregation. Moreover, the high-magnification SEM image demonstrates the individual Ti_3_C_2_ attached to the fabric (right panel of Fig. 3c). Figure 3d and Supplementary Fig. 3b shown the Ti_3_C_2_ elemental distribution and EDS spectrum, respectively on the CF, revealing that Ti, C, and O are evenly dispersed across the entire fabric surface. The XRD peak observed at angles 9.69° and 20.4° correspond to the (002) and (004) planes, respectively, representing Ti_3_C_2_ (Supplementary Fig. 4a)^32,33^.

Based on the above results, we suggest that FePS_3_@rGO composite could be promising electrode material for energy storage devices. To confirm the suitable potential window for SASC, FePS_3_@rGO composite and Ti_3_C_2_ were deposited on glassy carbon electrodes as a working electrode and the electrochemical performance was measured by a three-electrode configuration using 1 M H_2_SO_4_ as the electrolyte.

Supplementary Fig. 5a depicts the CV curves of the pristine FePS_3_ and varying amounts of rGO (0.6–2.4 wt%) in FePS_3_ composites at a scan rate of 50 mV s^−1^. A pair of wide redox peaks can be seen throughout the CV cycles, revealing that the capacitance is mostly obtained from the pseudocapacitance based on the reversible oxidation state of Fe atoms^13,14^. The CV integration area of the FePS_3_@rGO_0.6_ electrode is significantly larger than that of pristine FePS_3_. This is because the incorporation of rGO into the FePS_3_ can effectively enhance the interlayer space and reduce FePS_3_ restacking, resulting in a large surface area available for electrolyte ions. When the 1.8 wt% rGO is introduced into the FePS_3_, the relevant CV integration area reaches the maximum and provides the highest C_sp_ values. However, the CV integration area of FePS_3_@rGO_2.4_ is smaller than FePS_3_@rGO_1.8_ because the large amount of rGO can reduce the pseudocapacitive reaction and conductivity of the electrode. Thus, we decided to undertake a comprehensive electrochemical study with optimum FePS_3_@rGO_1.8_ composite. Supplementary Fig. 5b shows the CV curves of FePS_3_@rGO_1.8_ electrode at different scan rates from 10–100 mV s^−1^. The CV curve retains anodic and cathodic peaks without distortion at a high scan rate, implying an excellent rate capability and capacitive nature.

In Supplementary Fig. 5c, the GCD curves of the FePS_3_@rGO_1.8_ electrode at current densities ranging from 1 to 10 A g^−1^, revealed a non-symmetrical shape of charge/discharge curves. It was found that the discharge time was monotonically declined as a function of current density. This could be due to the low absorption of ions onto the surface of nanosheets at a rapidly changing potential. The FeS_3_@rGO exhibited a high C_sp_ of 175 F g^−1^ at 1.0 A g^−1^ and still maintained C_sp_ retention above 44.5% (78 F g^−1^) even at a high current density (Supplementary Fig. 5d). To further investigate the kinetics of ion transport of the pristine FePS_3_ and FePS_3_@rGO_1.8_ electrodes, electrochemical impedance spectroscopy (EIS) was performed (Supplementary Fig. 5e). The Nyquist plot exhibits lower equivalent series resistance (ESR) and interfacial charge-transfer resistance (R_ct_) of FePS_3_@rGO_1.8_ compared to pristine FePS_3_, indicating that the conductivity and ion transport of FePS_3_@rGO_1.8_ was increased after introducing rGO. Furthermore, the vertical slope of FePS_3_@rGO_1.8_ is much higher than pristine FePS_3_, implying that FePS_3_@rGO_1.8_ has a lower diffusion resistance^34,35^.

To meet the outstanding performance of the FePS_3_@rGO positive electrode, a suitable negative electrode is necessary to find a high-performance ASC device. Supplementary Fig. 5f–h depicts the electrochemical performance of Ti_3_C_2_. CV curves in Supplementary Fig. 5f show a pair of distinct redox peaks at 100 mV s^−1^, indicating a quick and reversible redox reaction of Ti atoms^36,37^. The GCD curves (Supplementary Fig. 5g) of Ti_3_C_2_ are nonlinear, showing its pseudocapacitive nature^38,39^. In comparison to C_sp_ of 153 F g^−1^ at a current density of 1.0 A g^−1^, the Ti_3_C_2_ retained 55% of its C_sp_ at high current density, proving its high rate capability (Supplementary Fig. 5h).

### Electrochemical performance evaluation

**Fig. 4:**
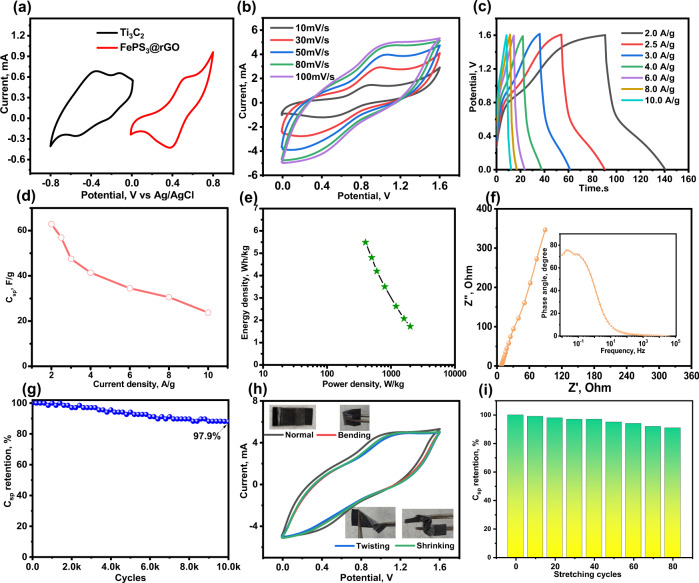
Electrochemical performance of Ti_3_C_2_/FePS_3_@rGO SASC. **a** CV curves of Ti_3_C_2_ and FePS_3_ at scan rate 50 mV s^−1^. **b** CV curves of Ti_3_C_2_/FePS_3_@rGO SASC at different scan rates. **c** GCD plots as function of current densities. **d** C_sp_ at different current densities. **e** Ragone plot: power density *versus* energy density. **f** Nyquist plot (inset display bode plot). **g** Long-term cycling stability. **h** CV curves of the device measured at various bending modes. **i** C_sp_ retention versus 30% stretching over 80 cycles.

We further fabricated SASC device employing Ti_3_C_2_ as the negative electrode and FePS_3_@rGO as the positive electrode in polymeric hydrogel electrolyte. The charge balance between Ti_3_C_2_ NS and FePS_3_@rGO electrodes to maximize the potential window of the Ti_3_C_2_/FePS_3_@rGO SASC is obtained by balancing the mass loading before device fabrication. The mass ratio of FePS_3_@rGO to Ti_3_C_2_ was maintained at 0.89 to charge both electrodes. Figure 4a shows the potential windows of the Ti_3_C_2_ (0 to −0.8 V) and FePS_3_ (0 to 0.8 V) at the scan rate of 50 mV s^−1^, demonstrating that each electrode stored a similar charge. Finally, a working window of up to 1.6 V is attained for the SASC, which can be delivered with higher capacitance, power, and energy density than the individual electrode devices. Figure 4b shows the CV curves of the Ti_3_C_2_/FePS_3_@rGO SASC at a scan rate of 10–100 mV s^−1^. It exhibits a stable operating window of 1.6 V as well as a pair of redox peaks. There was no significant distortion at a high scan rate of 100 mV s^−1^, indicating that the electrodes possess low resistance and rapid and reversible charge/discharge capabilities. In addition, the GCD curves of SASC at various current densities (2–10 A g^−1^) are approximately symmetric with a plateau that corresponds to the CV results (Fig. 4c). The C_sp_ values calculated from GCD plots as a function of current densities (Fig. 4d) show that the SASC exhibited a high gravimetric C_sp_ of 62.9 F g^−1^ at 1.0 A g^−1^. Notably, the SASC also demonstrated impressive rate performance with 23.7 F g^−1^ C_sp_ at a high current density of 10 A g^−1^.

The Ragone plot (Fig. 4e) shows the power (P) and energy (E) densities of the SASC. It exhibited a high E of 5.59 Wh kg^−1^ at P of 400 W kg^−1^ and retained E of 2.1 Wh kg^−1^ at P of 2 KW kg^−1^. A Ragone plot comparing the performance of Ti_3_C_2_/FePS_3_@rGO SASC with different reported supercapacitors is shown in Supplementary Table 1. Notably, E of SASC comparable to the pristine rGO, Ti_3_C_2_T_x_, NiPS_3_ and their composites. Whereas, SASC shows three fold higher power densities. These results indicate that SASC should be able compete with micro batteries in a wide range of health care applications. Further, EIS was used to estimate the charge transfer and internal resistance of the SASC device (Fig. 4f). The values of ESR and R_ct_ calculated from the equivalent circuit are 7.8 and 2.5 Ω, respectively. The low R_ct_ value can be ascribed to the high interfacial conductivity of both electrodes. Inset of Fig. 4f shows the phase angle of −72° at low frequency is very close to the ideal capacitors (−90°)^33,40^. Next, the cycling stability of SASC was conducted at a high current density (10 A g^−1^) for 10,000 charge/discharge cycles. As seen in Fig. 4g, C_sp_ retention after 10,000 cycles is 97.9% of its initial C_sp_, demonstrating outstanding cycling stability.

**Fig. 5:**
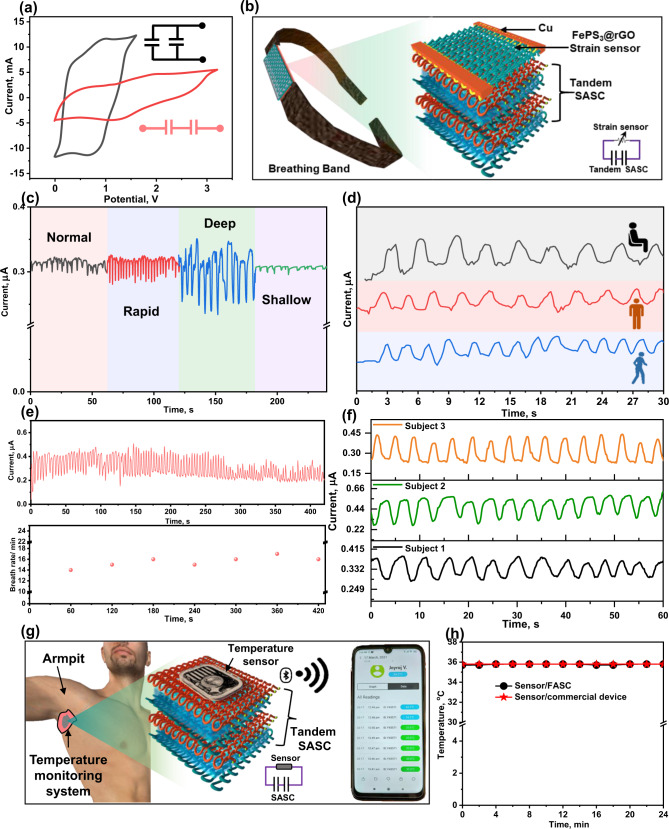
Remotely monitoring physiological parameters of human. **a** CV profile of the SASC connected in parallel and in series. **b** Schematic illustration of breathing band containing integrated FePS_3_@rGO strain sensor with tandem SASC. **c** Breathing signals recorded at normal, rapid, deep, and shallow breathings. **d** Breathing pattern as a function of activity such as sitting, walking, and running. **e** Multiple breathing cycles and corresponding breathing rate per minute. **f** Breathing rate of three volunteer subjects. **g** Integrated temperature sensor with tandem Ti_3_C_2_/FePS_3_@rGO SASC to monitor the user’s body temperature and wirelessly transmit data to the mobile phone. **h** Real temperature as a function of time as measured by the integrated temperature sensor operated by Ti_3_C_2_/FePS_3_@rGO SASC and bias of 1.5 V.

As studied above, the SASC exhibited a wide potential window, excellent capacitance, rate capability, cycling stability, and power density. To demonstrate the SASC as a portable power source in healthcare applications, it is important to investigate device mechanical properties and power output. Figure 4h displays that the CV curves are identical in different bending modes (i.e., bending, twisting, and shrinking), demonstrating the satisfactory mechanical stability and robustness of the SASC for realistic wearable applications. The CV profiles of the SASC under various applied tensile strains (Supplementary Fig. 6) exhibit negligible alterations, indicating that the device performance remains unchanged even when subjected to 30% tensile strain. Likewise, the electrochemical performance demonstrates outstanding durability when subjected to several stretching cycles. The stretching study of SASC resulted in 91% C_sp_ retention after 80 cycles (Fig. 4i). These findings demonstrate that our SASC can withstand high mechanical deformation while still delivering outstanding energy storage performance. The performance of this device was normal when compared to other ASC, but their exceptional mechanical features inspired the construction of a portable power source for the healthcare monitoring system. In real applications, the voltage and capacitance requirements are variable; hence, the SASCs should be coupled in parallel or in series to satisfy the needs. For example, when two SASCs are connected in series, the output voltage is increased to 3.2 V. While two SASCs are connected in parallel, the enclosed area of CV curves can be double that of a single SASC (Fig. 5a).

### Remotely monitoring physiological parameters

Further, we developed a wearable breathing band composed of the tandem SASC and FePS_3_@rGO-based stain sensor (Fig. 5b). This integrated system can detect human breathing in real-time. Fabrication details for the FePS_3_@rGO strain sensor are found in the supporting information. To investigate the FePS_3_@rGO suitability for usage in wearable strain sensor, the FePS_3_@rGO was coupled in a closed circuit with a red-light emitting diode (LED). The red LED glow faded dramatically when the FePS_3_@rGO was stretched, showing a change of electrical conductivity (or resistance) with stretching (Supplementary Movie 1). This ability to translate mechanical distortion into a detectable electrical signal could be used for strain sensing. SEM measurements were used to demonstrate how the morphology of FePS_3_@rGO strain sensor changes upon stretching. The digital photographs and SEM images of the FePS_3_@rGO strain sensor with and without strain are shown in Supplementary Fig. 7. We can observe that the contact between meandering loops remains when being expanded in the y-direction. Therefore, we assembled the FePS_3_@rGO strain sensor and SASC in the same direction.

To evaluate the stretch properties of the strain sensor, the difference in relative resistance was measured as a function of tensile strain (Supplementary Fig. 8a). The relative resistance is defined as *ΔR/R*_*0*_, where *ΔR* = *R* − *R*_*0*_ and *R*_*0*_ and *R* are the resistance before and after the applied strain, respectively^41^. Applied tensile strain is calculated by *ε* = *(L* − *L*_*0*_*)/L*_*0*_ *×* *100*, where *L*_*0*_ is initial length and *L* is length after being stretched^42^. It was found that the FePS_3_@rGO strain sensor resistance increased linearly with a corresponding gauge factor of 38.7 (*R*^*2*^ = 0.96) at 40% strain. The obtained gauge factor value was superior to that previously reported for some of the 2D materials and carbon-coated textile strain sensors^43–46^. The repeatability of the FePS_3_@rGO strain sensor was then examined using step and hold strain cycles, and the results are displayed in Supplementary Fig. 8b. The sensor resistance achieved a steady plateau and stayed unchanged during the holding and repeating strain cycles. Then, a response time for the stretching and releasing process was determined to be ~2.38 s at 1% strain (Supplementary Fig. 8c).

Based on satisfactory results of SASC and strain sensor were integrated into one textile and fabricated breathing monitoring band. Typically, the normal breathing rate for the adult is around 16–20 breaths per minute^47,48^. When individuals are infected with viruses such as COVID 19, they can get severe breathing syndrome and have trouble breathing normally^49,50^. In this situation, remotely monitored breathing is required to provide real-time measurements and alerts to quick action, particularly for those with moderate symptoms^51,52^. Current breathing monitoring systems are too costly and inconvenient to be used for prolonged periods. As a result, a low cost and lightweight breathing monitoring system is proposed. The fabricated breathing monitoring band is wrapped around the middle part of the abdomen, which can continuously monitor breath cycles of the individual and relay data to a mobile device *via* wireless communication (Supplementary Figs. 9a and 10a). As we know, inhalation and exhalation comprise one complete breathing cycle. When a user inhales and exhales, the abdomen expands and contracts, respectively, causing the FePS_3_@rGO strain sensor to stretch and release, resulting in electrical (or resistance) signals. Using the breathing monitoring band, a real-time breathing pattern of an individual was obtained in 60 s with four distinct breathing states: normal, fast, slow, and shallow (Fig. 5c). The rate obtained for normal breathing is around 14 breaths per minute (bpm) while the rate for fast breathing is 23 bpm. However, slow and shallow breathing states calculated rates are 12 and 9 bpm, respectively.

Further, breathing patterns were monitored in real-time when sitting, walking, and running (Fig. 5d). The current signal obtained by the breathing band can clearly differentiate between the various physical activities. For instance, the number of breathing cycles when running (~26 bpm) is higher than when walking (~20 bpm) and sitting (~15 bpm). A long-term breathing cycle and corresponding calculated breathing rate is presented in Fig. 5e. It can be clearly seen that the current signals of repeated inhalations and exhalations remain steady with a breathing rate of 14–16 bpm. We also measured the breathing rate of three male volunteers. The current responses generated by their breathing cycles are shown in Fig. 5f. Subjects’ 1, 2, and 3 average breathing rate was 13, 14, and 16 bpm, respectively, which demonstrates good reliability of the breath monitoring system. All of these demonstrations indicate that our integrated FePS_3_@rGO strain sensor is capable of continuously measuring breathing cycles in real time. Supplementary Movie 2 shows the real-time remote sensing performance of the integrated FePS_3_@rGO strain sensor in response to repeated breathing strain when connected to a wireless transmitter: the response is quick and repeatable. The signal can alert the user or a healthcare facility if a situation emerges when the breathing rate exceeds or goes below the typical range. As a result, medical personnel can take the necessary actions such as dispatching an ambulance to the patient’s location.

Following the satisfactorily integrated FePS_3_@rGO strain sensor performance, a wearable temperature monitoring system was further developed that can detect abnormal body temperatures (such as fever) and be worn on a smartphone *via* wireless communication. Figure 5g shows the temperature sensor was linked with the tandem SASC. The Bluetooth module enables the recorded temperature sensor data to be sent to a smartphone app (Supplementary Figs. 9b and 10b). Bluetooth have been used widely to transfer data from medical devices to common consumer devices (smartphones, smartwatches and laptops). Moreover, Bluetooth is relatively biocompatible, user friendly and very affordable^53^.

The self-discharge profile of the tandem Ti_3_C_2_/FePS_3_@rGO SASC (Supplementary Fig. 6b) exhibits ~53.0% (1.7 V) of the initial charge potential after 2000s of self-discharge. This result demonstrates that the temperature sensor can be driven for long periods by a tandem SASC device. Figure 5h depicts the recorded body temperature as a function of time, demonstrating that the Ti_3_C_2_/FePS_3_@rGO SASC can generate a steady power supply to the integrated temperature sensor. Likewise, a constant voltage of 1.5 V (control experiment) was used to measure real body temperature by the integrated temperature sensor. The obtained results of the temperature sensor driven by Ti_3_C_2_/FePS_3_@rGO SASC and constant voltage demonstrated the possibility of using SASC to power the temperature sensor instead of a commercial energy source. Real-time temperature detection in the human body (early identification of fever) aids in illness mitigation. Most importantly, this wearable system assists in identifying whether or not a user is infected with SARS-CoV-2 or other viroid diseases and allows for a quick response.

In summary, we successfully developed a wearable health monitoring system using integrated sensors with SASC to monitor real-time physiological parameters of remote individuals for the early identification of viral infection or emergencies. The spray-coating deposition method was used to prepare Ti_3_C_2_ and FePS_3_@rGO-based stretchable electrodes and the Ti_3_C_2_/FePS_3_@rGO SASC was assembled using polymer gel electrolyte. The assembled SASC device exhibits high electrochemical performance in terms of C_sp_, cycling stability, power density, rate capability, bending, and stretching stability. Further, FePS_3_@rGO-based energy storage device and strain sensors to be fabricated as a flexible and stretchable power source and a sensor for monitoring real-time breathing cycles. We successfully monitored live breathing patterns as a function of activity in volunteers. Lastly, we integrated the Ti_3_C_2_/FePS_3_@rGO SASC with a temperature sensor that can be affixed to a user’s skin and accurately monitor a person’s body temperature in real time and wirelessly relay data to a smartphone. The wireless devices developed in this work, can be used in the emergence room to monitor of various infectious (viral/bacteria) diseases, especially if hospitals need to isolate infected patients to prevent the spreading of pathogens to health care personnel. Moreover, this study paves the way for the development of innovative wearable e-health monitoring systems based on flexible and stretchable energy storage devices.

## Methods

### Materials

Lithium tetrafluoroborate (LiBF_4_) and pluronic polymer (F108) were purchased from Sigma Aldrich. Conductive carbon ink was obtained from DuPont. A wearable temperature sensor was purchased from Amazon in the Czech Republic. Stretchable cotton fabric was purchased at a local shop in Prague, Czech Republic.

### Preparation of FePS_3_@rGO and Ti_3_C_2_ based fabric electrodes

Cotton fabric (CF) was chosen as the substrates for coating FePS_3_@rGO and Ti_3_C_2_ materials due to their abundance of oxygen-containing functional groups. In addition, fabric has higher hydrophilicity than synthetic textiles, which aids in functional material adsorption. Carbon ink was used to make fabric conductive before active 2D materials were deposited on its surface. The carbon ink coated stretchable fabrics were dried at 50 °C for 30 min in the electronic oven. Afterwards, the FePS_3_@rGO colloidal solution was sprayed onto the fabric substrate. Additionally, four different FePS_3_@rGO suspensions were made with rGO weight percentages of 0.6, 1.2, 1.8 and 2.4 in the mixture donated as FePS_3_@rGO_0.6_, FePS_3_@rGO_1.2_, FePS_3_@rGO_1.8_, and FePS_3_@rGO_2.4_, respectively. The height between the spray nozzle and CF substrate remains constant during the spray-coating process. A similar process was used to make Ti_3_C_2_ coated fabric. Finally, all electrodes were dried at 55 °C to ensure water elimination.

### Fabrication of SASC and integrated healthcare system

*Ti*_*3*_*C*_*2*_*/FePS*_*3*_*@rGO SASC:* The SASC was fabricated by sandwiching the F108@LiBF_4_ gel electrolyte between the positive (FePS_3_@rGO-coated fabric) and negative (Ti_3_C_2_ coated fabric) electrodes. The F108@LiBF_4_ gel electrolyte is made up of 35 % w/w pluronic polymer (Pluronic®-F108, PEO_122_-PPO_50_-PEO_133_) dissolved in 1 M LiBF_4_ aqueous solution.

*Wearable temperature monitoring system*: The temperature sensor was connected with tandem Ti_3_C_2_/FePS_3_@rGO SASC using copper tap to assemble the integrated temperature sensor. The temperature sensor is directly attached to the user’s armpit and monitors real-time body temperature while wirelessly transmitting data to the mobile phone.

*Wearable breath monitoring system*: The FePS_3_@rGO strain sensor and tandem Ti_3_C_2_/FePS_3_@rGO SASC were interconnected using silver ink and copper tap. The energy stored in the Ti_3_C_2_/FePS_3_@rGO SASC can be used to power the strain sensor.

### Materials characterizations and electrochemical measurements

The morphology and structural properties of the FePS_3_ and Ti_3_C_2_ were analyzed using scanning electron microscopy (SEM, JEOL 7600F, Japan), transmission electron microscopy (TEM), energy-dispersive spectroscopy (EDS, SDD detector X-MaxN 80TS), X-ray powder diffraction (XRD, Bruker, D8, Germany) and Brunauer-Emmett–Teller (BET, Quantachrome Instrument) method. The electrochemical performance of SASC was evaluated by cyclic voltammetry (CV), galvanostatic charge-discharge (GCD) and electrochemical impedance spectroscopy (EIS) measurements on an electrochemical workstation (Autolab PGSTAT204, Netherlands) using two and three-electrode configurations. Three electrode configurations, glassy carbon coated FePS_3_@rGO, Ag/AgCl and platinum disk were used as working, reference and counter electrodes, respectively in 1 M H_2_SO_4_ aqueous solution. EIS measurements were performed in the frequency range between 10^−2^ and 10^5 ^Hz with AC amplitude of 10 mV and open-circuit voltage.

The charge balancing mechanism was used to calculate the loading mass of active materials on negative and positive electrodes using Eq. (1). The gravimetric specific capacitance (C_sp_, F g^−1^), power density (P, W kg^−1^) and energy density (E, Wh kg^−1^) were calculated based on the total mass of the active materials according to the following Eqs. (2), (3) and (4).

1 $$\frac{{m_ + }}{{m_ - }} = \frac{{C_ - \times \Delta V_ - }}{{C_ + \Delta V_ + }}$$

2 $$C_{SP} = I\Delta t/m\Delta V$$

3 $$E = C_{SP}(\Delta V)^2/8$$

4 $$P = E/\Delta t$$

Where, Δt, *V, I, and m are the* total discharge time, operating voltage window, discharge current and total mass of the two electrodes, respectively.

## Supplementary information

Supplementary information

Video S1

Video S2

## Data Availability

The data that support the findings of this study are available from the corresponding author upon reasonable request.
