# Supplementary material for: Telemedicine platform for health assessment remotely by an integrated nanoarchitectonics FePS3/rGO and Ti3C2-based wearable device
Source: Npj Flex Electron. 2022 Aug 15;6(1):73. doi: 10.1038/s41528-022-00208-1 (PMC9376913; doi:10.1038/s41528-022-00208-1)
Supplement: Supplementary file 1 — Supplementary information [file 41528_2022_208_MOESM1_ESM.pdf]

Supplementary Information

Telemedicine Platform for Health Assessment Remotely by an Integrated Nanoarchitectonics $FePS_3$/rGO and Ti_3C_2-based Wearable Device

Jayraj V. Vaghasiya[1], Carmen C. Mayorga-Martinez[1], Martin Pumera[1,2,3,4]*

[1]Center for Advanced Functional Nanorobots, Department of Inorganic Chemistry, Faculty of Chemical Technology, University of Chemistry and Technology Prague, Technická 5, 166 28 Prague, Czech Republic

[2]Energy Research Institute@NTU (ERI@N), Research Techno Plaza, X-Frontier Block, Level 5, 50 Nanyang Drive, 637553 Singapore, Singapore

[3]Faculty of Electrical Engineering and Computer Science, VSB - Technical University of Ostrava, 17. listopadu 2172/15, 70800 Ostrava, Czech Republic

[4]Department of Medical Research, China Medical, University Hospital, China Medical University, No. 91 Hsueh-Shih Road, Taichung 40402, Taiwan

E-mail: pumera.research@gmail.com

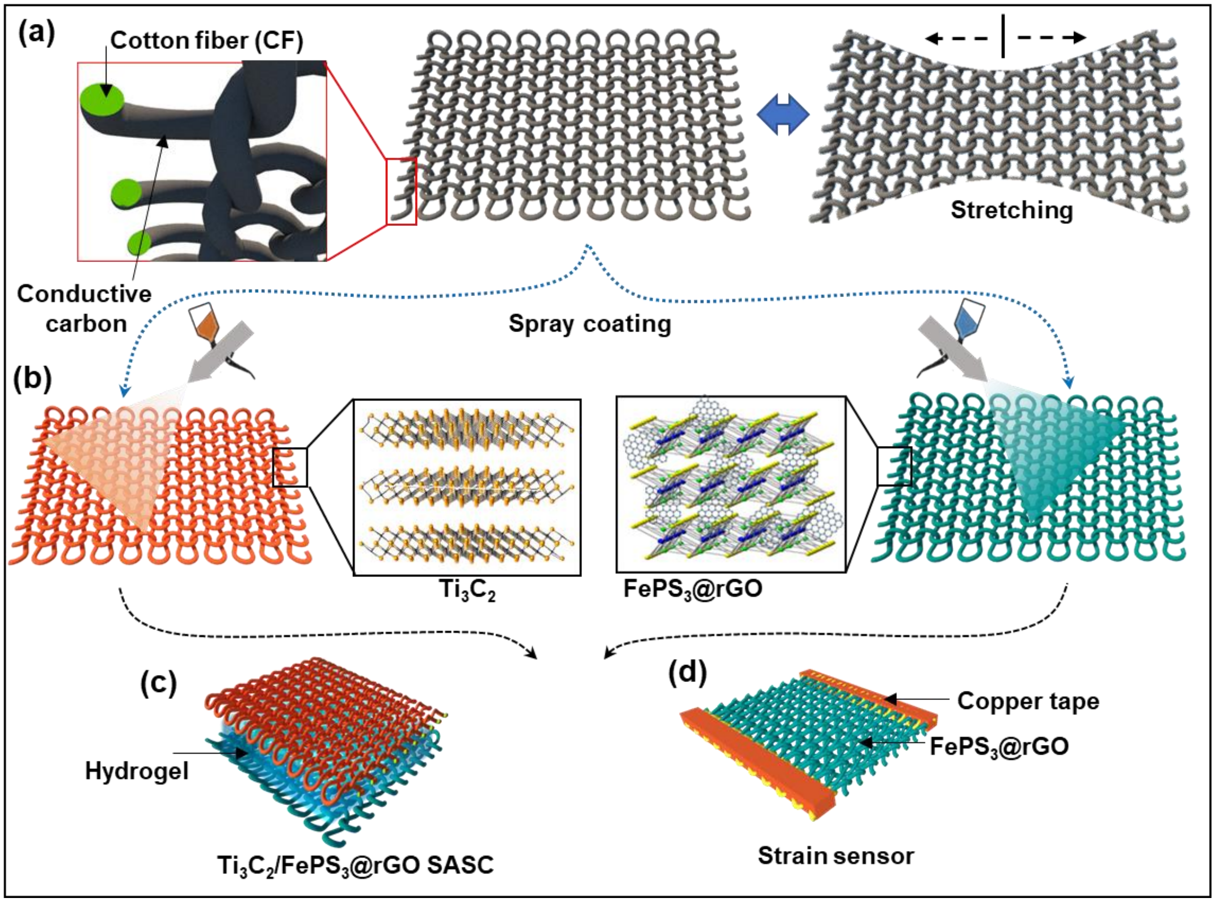

Supplementary Figure 1. A fabrication process of Ti_3C_2/$FePS_3$@rGO SASC: **(a)** Schematic depiction of the conducting cotton fabric (CF) in normal and stretch mode, **(b)** spray-coating of $FePS_3$@rGO and Ti_3C_2 on fabric, **(c, d)** architecture of the Ti_3C_2/$FePS_3$@rGO SASC and $FePS_3$@rGO based strain sensor, respectively.

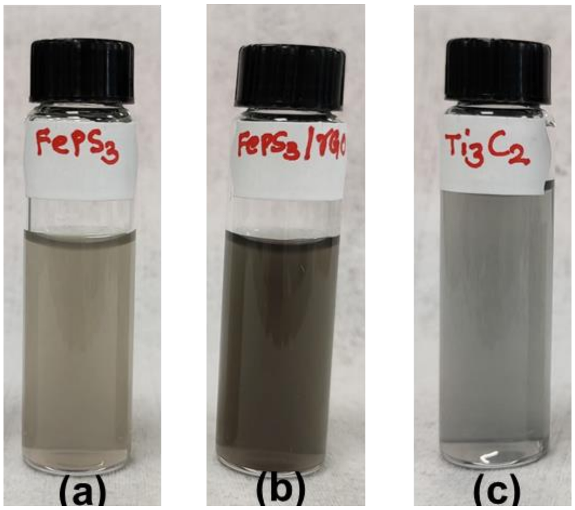

Supplementary Figure 2. Colloidal solution of **(a)** $FePS_3$, **(b)** $FePS_3$@rGO, and **(c)** Ti_3C_2.

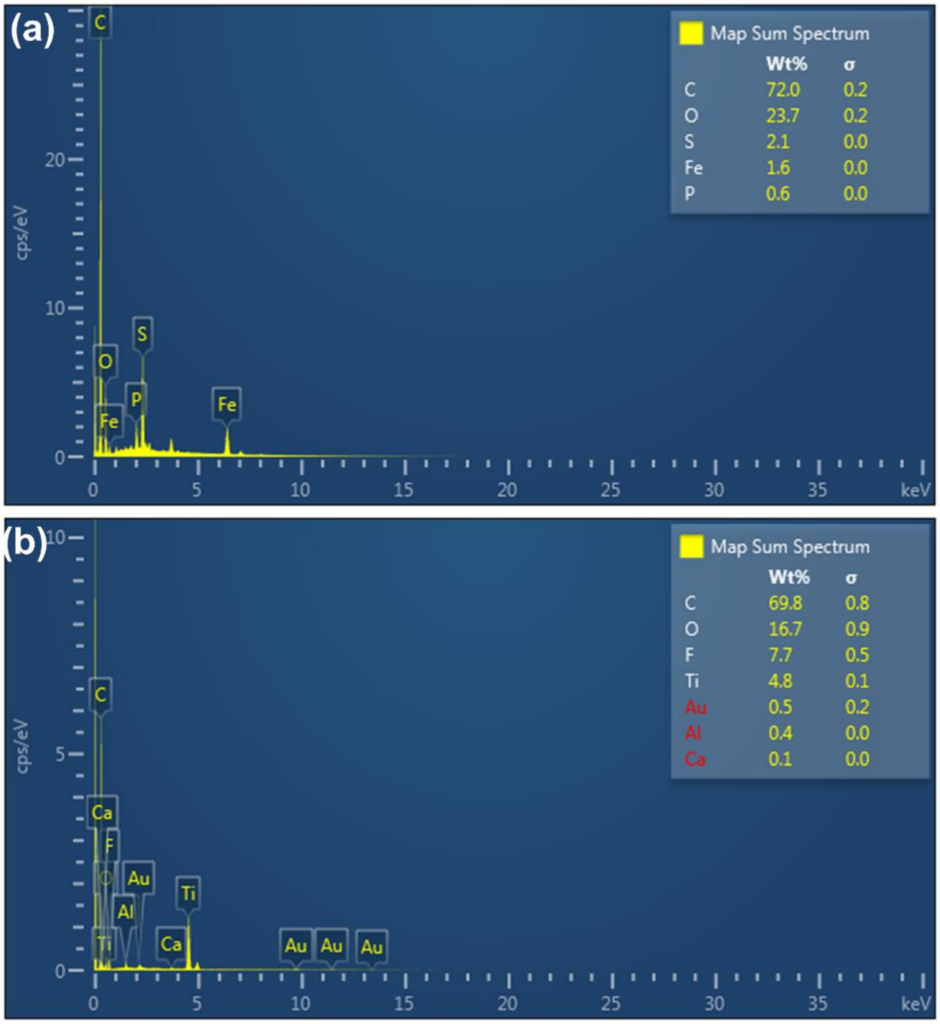

Supplementary Figure 3. EDS spectra of $FePS_3$@rGO (a) and Ti_3C_2 (b) coated fabrics.

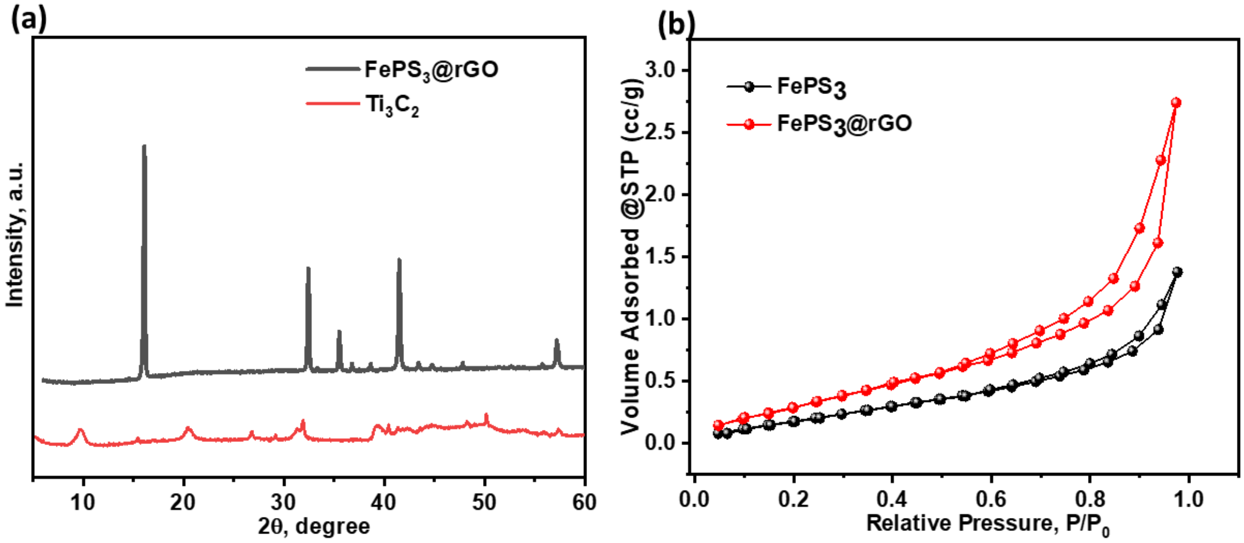

Supplementary Figure 4. **(a)** XRD pattern of $FePS_3$ and Ti_3C_2, and **(b)** nitrogen adsorption-desorption isotherm of pristine $FePS_3$ and $FePS_3$@rGO.

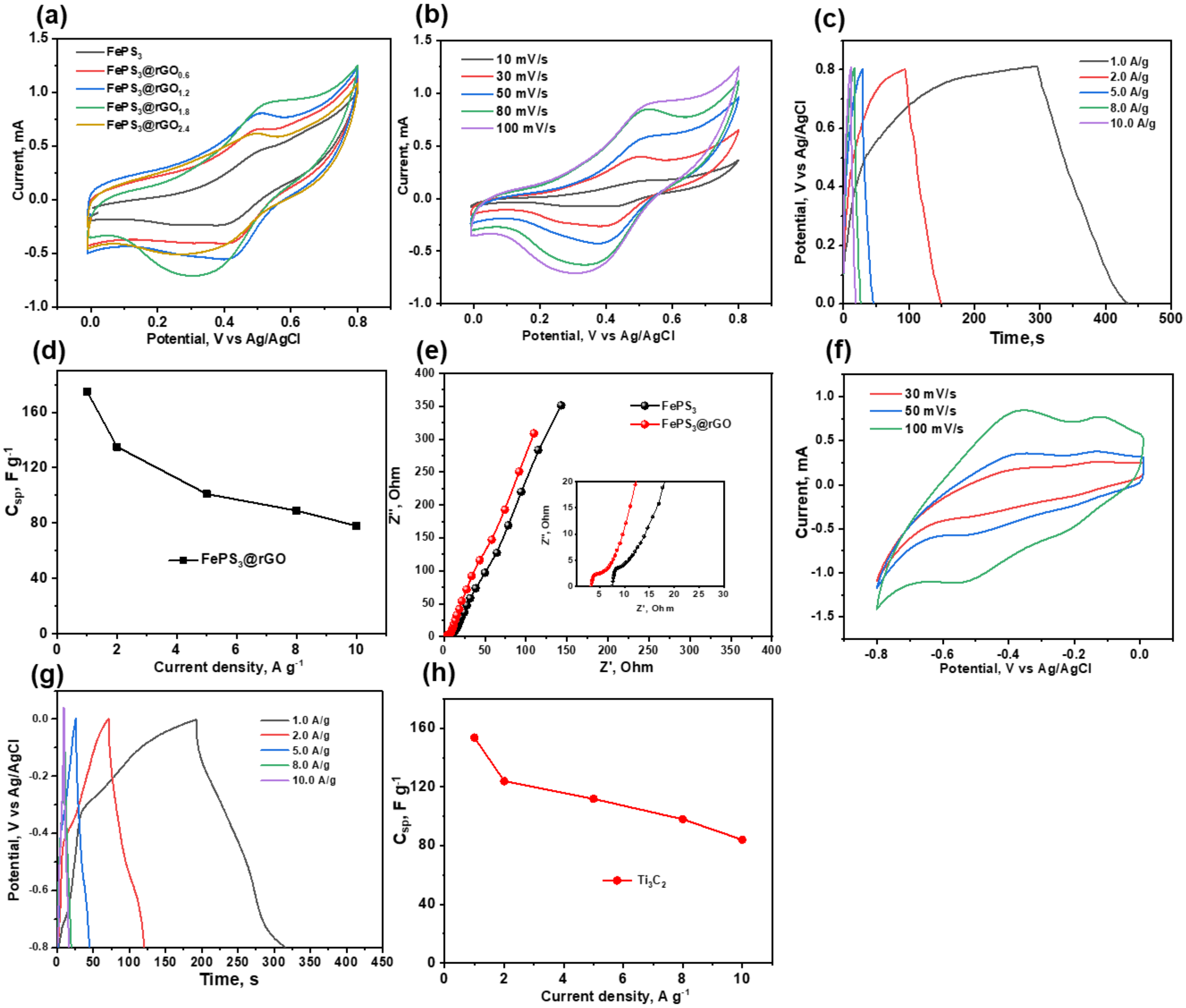

Supplementary Figure 5. Electrochemical performance in the three-electrode system; **(a)** CV curves of pristine $FePS_3$ and different amounts of rGO in $FePS_3$ electrodes at a scan rate of 50 mV/s; **(b)** CV curves of $FePS_3$@$rGO_{1.8}$ at different scan rates; **(c)** GCD plots of $FePS_3$@$rGO_{1.8}$ as a function of current densities; **(d)** C_{sp} versus current densities of $FePS_3$@$rGO_{1.8}$ electrode; **(e)** Nyquist plots of pristine $FePS_3$ and $FePS_3$@$rGO_{1.8}$ electrodes (inset shows a magnification of high-frequency region); **(f)** CV curves of Ti_3C_2 at different scan rates; **(g)** GCD plots of Ti_3C_2 as a function of current densities; **(h)** C_{sp} versus current densities of Ti_3C_2 electrode.

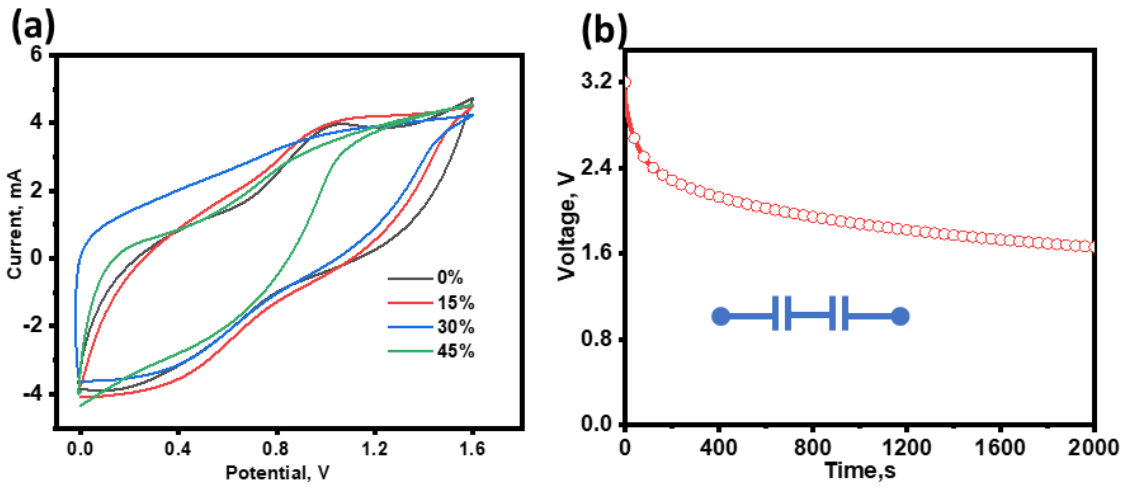

Supplementary Figure 6. **(a)** CV curves measured at a scan rate of 50 mV s^{-1} under different tensile strains; **(b)** self-discharge curve of two serially connected SASC.

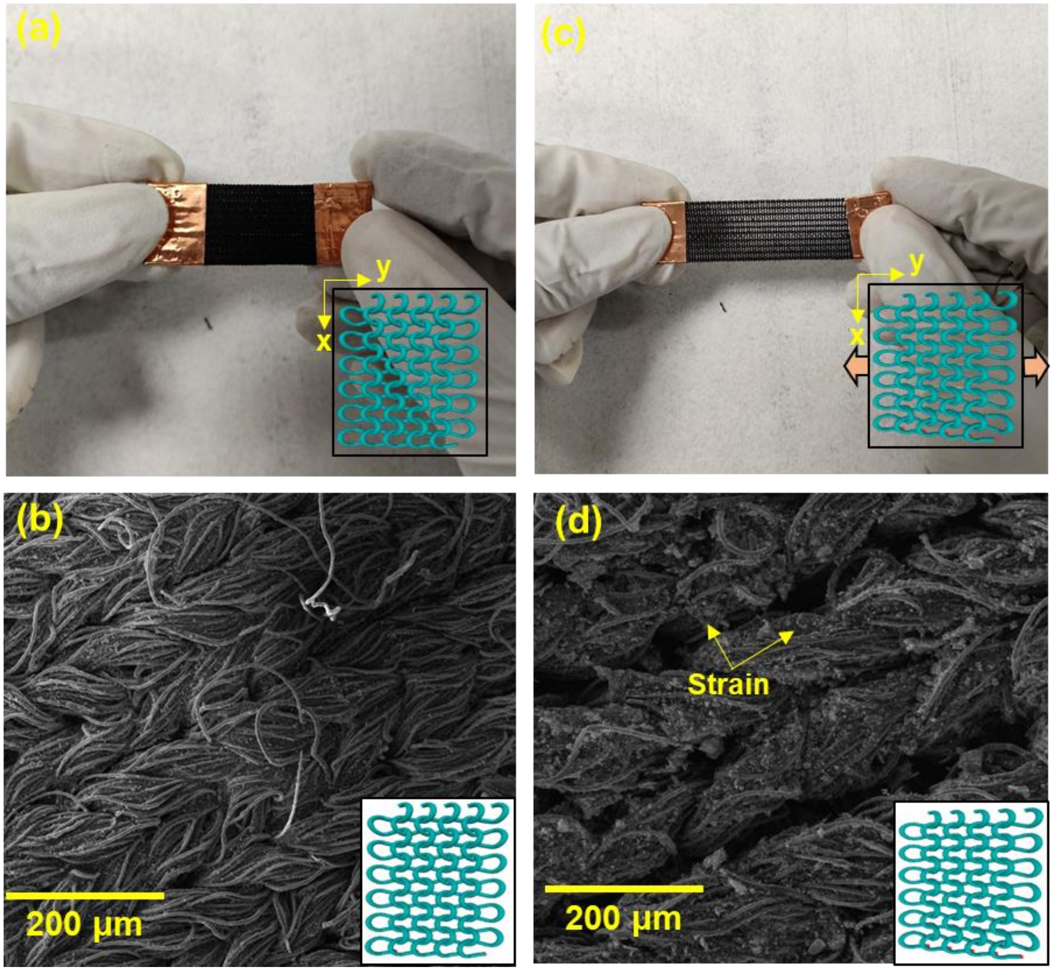

Supplementary Figure 7. (a, b) Digital photograph and loop architecture of $FePS_3$@rGO strain sensor without strain and corresponding SEM image. **(c, d)** Digital photograph and loop architecture of $FePS_3$@rGO strain sensor stretched in the y-direction and corresponding SEM image.

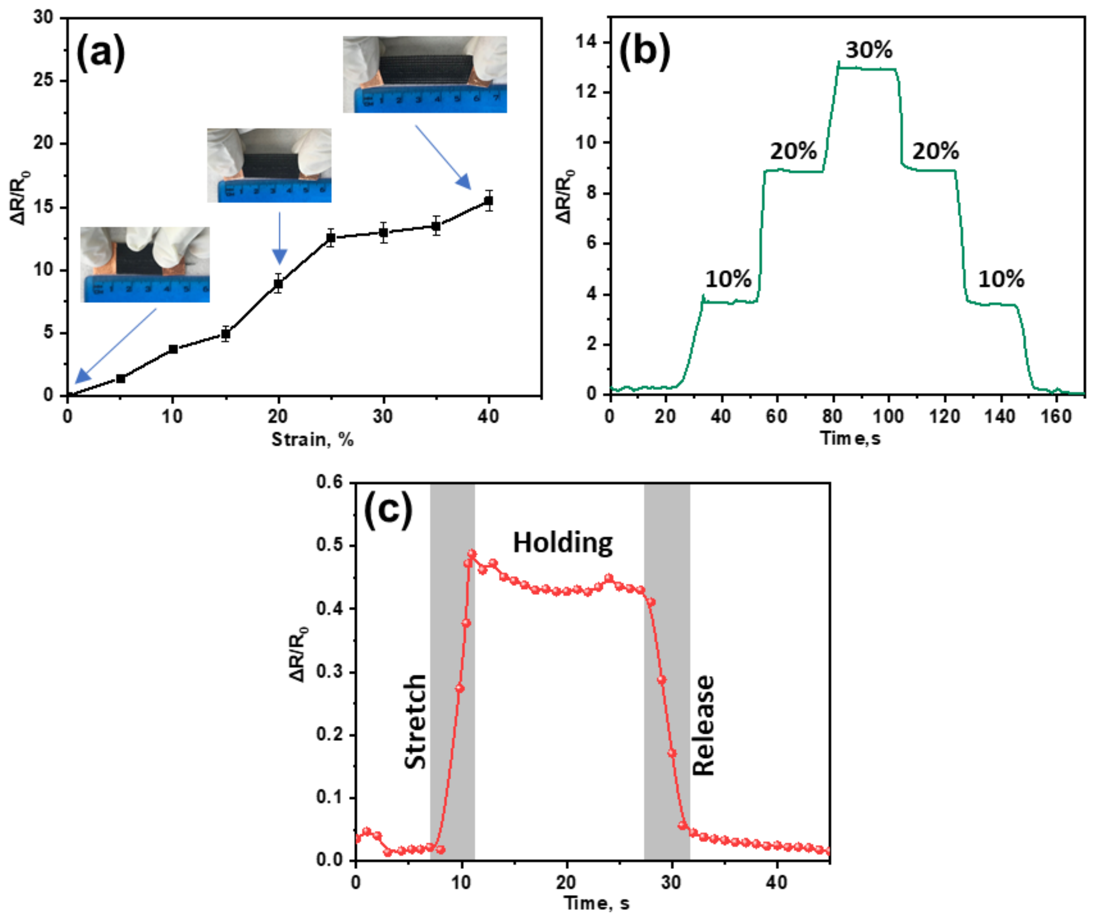

Supplementary Figure 8. **(a)** Relative resistance versus strain response curves of $FePS_3$@rGO strain sensor; **(b)** repetitive measurements of the variation in the relative resistance as a function of a strain of 10, 20 and 30%; **(c)** response curve with an applied strain of 1%.

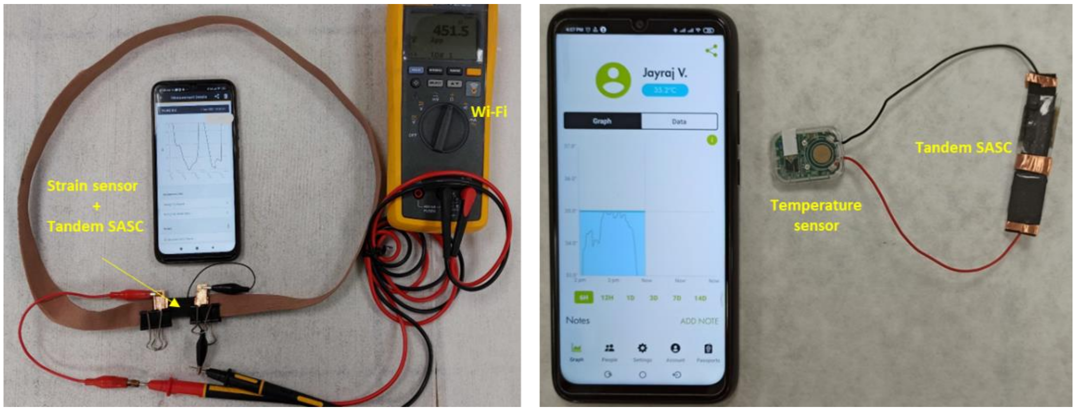

Supplementary Figure 9. Digital photograph of an integrated $FePS_3$@rGO strain sensor (left side image) and temperature sensor (right side image) with series-connected Ti_3C_2/$FePS_3$ SASC and wirelessly relay data to a mobile phone.

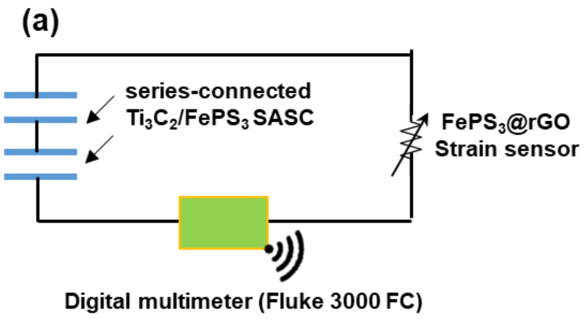

Supplementary Figure 10. The schematic illustration of the electric circuit of the integrated strain sensor **(a)** and temperature sensor **(b)**.

Supplementary Table 1. The gravimetric capacitance, energy density and power density of reported supercapacitors. (NA: not available)

Electrode	Electrolyte	C_{sp}, F/g	E, Wh kg^{-1}	P, W kg^{-1}	Ref.
rGO	H_3PO_4/PVA	56.11 (at 5 mV s^{-1})	1.95	159.8	1
rGO-PEDOT/PSS	H_3PO_4/PVA	132 (at 5 mV s^{-1})	2.83	3589	1
co-doped GO aerogels	H_2SO_4/PVA	62 (at 5 mV s^{-1})	8.65	1600	2
$NiPS_3$	3 M KOH	61.3 (at 1.0 A g^{-1})	19.2	750	3
$Ti_3C_2T_x$//rGO	Organic	48 (NA)	8	NA	4
$Ti_3C_2T_x$/MWCNT	Organic	7 (NA)	3	NA	4
MnO_2/Ti_3CT_x-ar	KOH	210 (at 0.1 A g^{-1})	5.47	700	5
Ti_3CT_x_Ar	KOH	35 (at 0.1 A g^{-1})	2.19	700	5
rGO	Ionic liquid	80.4 (at 0.1 A g^{-1})	11.2	NA	6
LT-$Ti_3C_2T_x$	3M KCl	467 (at 5 A g^{-1})	5.67	589	7
AC//Go@WO_3	H_2SO_4/PVA	465 (at 1 A g^{-1})	27	6000	8
AC//rGO/$NiSe_2$	3M KOH	114 (at 1 A g^{-1})	41	842	9
HRG//m-WO_3	1 M H_2SO_4	389 (at 0.5 A g^{-1})	93	500	10
Ti_3C_2//$FePS_3$@rGO	F108@$LiBF_4$	62.9 (at 1.0 A g^{-1})	5.59	400	This work
Ti_3C_2//$FePS_3$@rGO	F108@$LiBF_4$	62.9 (at 1.0 A g^{-1})	2.1	2000	

Supplementary Movie 1. Shows the resistivity as a function of stretch. The $FePS_3$@rGO-based strain sensor is connected in a closed circuit with a red LED.

Supplementary Movie 2. Remotely monitoring breathing cycles. An integrated $FePS_3$@rGO-based strain sensor and SASC fixing on the volunteer's abdomen.

Supplementary References

1. Liu, Y. et al. High-Performance flexible all-solid-state supercapacitor from large free-standing graphene-PEDOT/PSS films. *Sci. Rep.*, 5, 17045 (2015).

2. Wu, Z. S. et al. Three-dimensional nitrogen and boron Co-doped graphene for high-performance all-solid-state supercapacitors. *Adv. Mater.*, **24,** 5130-5135 (2012).

3. Asif, A. H. et al. Heterogeneous activation of peroxymonosulfate by Co-doped Fe_2O_3 nanospheres for degradation of p-hydroxybenzoic acid. *J. Colloid Interface Sci*. **616,** 401-402 (2022).

4. Navarro-Suárez, A. M. et al. Development of asymmetric supercapacitors with titanium carbide-reduced graphene oxide couples as electrodes. *Electrochim. Acta* **259,** 752-761 (2018).

5. Rakhi, R. B., Ahmed, B., Anjum, D. & Alshareef, H. N. Direct chemical synthesis of MnO_2 nanowhiskers on transition-metal carbide surfaces for supercapacitor applications. *ACS Appl. Mater. Interfaces* **8,** 18806-18814 (2016).

6. Rath, T. & Kundu, P. P. Reduced graphene oxide paper based nanocomposite materials for flexible supercapacitors. *RSC Adv.*, **5,** 26666-26674 (2015).

7. Pan, X., Shinde, N. M., Lee, M., Kim, D., Kim, K. H. & Kang, M. Controlled nanosheet morphology of titanium carbide Ti_3C_2Tx MXene via drying methods and its electrochemical analysis. *J. Solid State Electrochem.* **24,** 675-686 (2020).

8. Nayak, A. K., Das, A. K. & Pradhan, D. High performance solid-state asymmetric supercapacitor using green synthesized graphene-WO_3 nanowires nanocomposite. *ACS Sustainable Chem. Eng.* **5,** 10128 –10138 (2017).

9. Gu, Y. et al. N-doped reduced graphene oxide decorated $NiSe_2$ nanoparticles for high-performance asymmetric supercapacitors. *J. Power Sources* **425,** 60-68 (2019).

10. Ashraf, M. et al. A High-Performance asymmetric supercapacitor based on tungsten oxide nanoplates and highly reduced graphene oxide electrodes. *Chem. Eur. J.* **27,** 6973-6984 (2021).
